# OMWare: a tool for efficient assembly of genome-wide physical maps

**DOI:** 10.1186/s12859-016-1099-1

**Published:** 2016-07-25

**Authors:** Aaron R. Sharp, Joshua A. Udall

**Affiliations:** College of Life Sciences, Brigham Young University, Provo, UT 84602-2400 USA

## Abstract

**Background:**

Physical mapping of DNA with restriction enzymes allows for the characterization and assembly of much longer molecules than is feasible with sequencing. However, assemblies of physical map data are sensitive to input parameters, which describe noise inherent in the data collection process. One possible way to determine the parameter values that best describe a dataset is by trial and error.

**Results:**

Here we present OMWare, a tool that efficiently generated 405 *de novo* map assemblies of a single datasets collected from the cotton species *Gossypium raimondii*. The assemblies were generated using various input parameter values, and were completed more efficiently by re-using compatible intermediate results. These assemblies were assayed for contiguity, internal consistency, and accuracy.

**Conclusions:**

Resulting assemblies had variable qualities. Although highly accurate assemblies were found, contiguity and internal consistency metrics were poor predictors of accuracy.

## Background

Massively parallel sequencing (MPS) has and will continue to produce tremendous biological insights [1]. However, the ability to answer certain genomic questions is dependent on read length, and in some cases, the most commonly available read lengths are shorter than what is required [2]. For example, read length may limit the robustness of de novo genome assembly [3]. Single molecule, high molecular weight (HMW) DNA sequencing by PacBio has had success producing significantly longer read lengths than many other technologies [4], but even their impressive maximum read length, 40 kb, may still be too short to answer some questions regarding genomic structural variants [5]. Until sequencing technologies are able to characterize longer molecules, alternative methods for HMW DNA assembly are required. Restriction fragment length analysis has long been a preferred method for analyzing longer DNA molecules [6–8].

Recent technical developments commercialized by the company BioNano Genomics (BNG) increased throughput for this type of long molecule characterization. Their method uses modified restriction enzymes to incorporate single-strand breaks at restriction sites, which are then labeled by using polymerase to incorporate fluorescent nucleotide analogs. Labeled sample is loaded into an array of nanofabricated channels that linearize the DNA. Waves of DNA can be loaded into the channels and imaged with a high-powered microscope and high-resolution camera. Individual molecules are assembled based on shared patterns of restriction sites into representations of the entire genome.

As with any single molecule technology, there is significant noise in the raw data. Sources of noise in this type of mapping include limitations in camera resolution, enzyme efficiency (particularly in the presence of contaminants), and non-uniform behavior of fluorescent molecules and the DNA duplex [9]. Additionally, depending on genome size and complexity, restriction fragment length patterns may be similar at different genomic loci by chance. Successful assembly algorithms must compensate for this noise in order to reconstruct accurate models of chromosomes. These algorithms incorporate noise compensation measures such as fuzzy matching for lengths between restriction sites, modeling enzyme error probabilities, and requiring whole molecule alignments that are long and similar enough to be unlikely results of chance alone [9]. These compensating measures rely in large part on descriptions of the data error profile, which are provided by the user as input parameters. Therefore, optimum assembly requires that a user select appropriate input parameters.

There are methods for empirical estimation of error profiles, many of which rely on significant genomic resources. For example, BNG provides software that maps a random subset of molecules to a reference genome sequence assembly, and selects error parameters that maximize both the number of molecules that align, and the goodness of fit for those alignments. However, this method depends on a highly contiguous sequence assembly for the organism of interest, which might not be available. One potential alternative for selecting accurate parameters is trial and error. Using a variety of input parameters yields a variety of assemblies, from which an optimal solution might be chosen.

Trial and error is a computationally expensive strategy. In order to be feasible, it should minimize redundant calculations and re-use intermediate results wherever possible. BioNano Genomics produces their own software for *de novo* assembly, which, internally, can make use of intermediate results. However, the user interface for that software makes result re-use impractical. Therefore, to test the effectiveness of the trial and error strategy, a new interface had to be developed.

We approached the problems of short read limitations, noise in physical map data, and the computational intensity of the trial and error strategy using a specific dataset. *Gossypium raimondii* is a cotton species that is the closest living relative to one of the subgenome progenitors of the agriculturally significant allopolyploid, *Gossypium hirsutum* [10]. *Gossypium raimondii* has a high quality reference genome sequence assembly that was created using MPS, as well as genetic and traditional physical maps [11]. An extended abstract describing a portion of this work has been published previously [12].

## Methods

### Mapping high molecular weight DNA molecules

Young leaf tissue from several *Gossypium raimondii* plants was flash frozen in liquid nitrogen and shipped on dry ice to Kansas State University, a Certified Service Provider for long-range DNA mapping with BNG’s technology. They performed HMW DNA extraction according to a proprietary protocol that includes physical disruption of the cell wall, polyphenol isolation with PVP, and embedding of unlysed nuclei in agarose gel to prevent DNA shearing. DNA molecules were subjected to sequence-specific, single-strand nicking at sites recognized by two modified restriction enzymes, Nt.BspQ1 and Nt.BbvCl, simultaneously. These enzymes were selected by simulating digestion of the reference genome sequence assembly [11] with a variety of enzymes, and selecting the enzyme or pair of enzymes that gave the expected label density closest to 11 nicks per 100 kilobase-pairs (kbp). Restriction sites were labeled with fluorescent nucleotide analogs, which were incorporated by Taq polymerase, and the DNA backbone was stained with the non-specific, intercalating dye, YOYO-1. Finally, labeled, stained DNA molecules were linearized by physical constriction in nanoscopic channels, immobilized with an electric current, and imaged with a high-powered microscope and high-resolution camera. Software provided by BNG converted raw images into digital representations of individual molecules.

### Parameter estimation by trial and error

The user interface provided by BNG allows the user to specify a number of input parameters that are known to affect map assembly algorithms (see [13, 14]). A **significance threshold** for accepting pairwise molecule alignments is an assumption about genome complexity, which frequently, but not necessarily, scales with genome size. It is an indication of how probable an alignment between two molecules is expected to occur because of random chance instead of a common genomic locus. **False positive** and **false negative** label rates explain, respectively, the density of observed labels found at locations other than the expected restriction sites, and the proportion of restriction sites that are not labeled, due to enzyme inefficiency. It is an assumption of the algorithm that false positive labels and false negative labels are distributed randomly throughout the genome. **Minimum molecule length** and **minimum labels per molecule** are not assumptions about the data error profile, or the genomic complexity. Rather, they represent a compromise between the amount of data included and the reliability of each data point, where longer, more label-dense molecules are more reliable. Additionally, the user interface has multiple parameters to describe variance in observed distances between labels compared to actual restriction site distributions, which is caused by molecule stretching and non-uniform stain behavior, as well as options relevant to the assembly refinement processes (see [15]). Although all of these parameters do not apply uniformly to all of the steps in the assembly process, the user interface only allows a single designation for each.

We designed and wrote Python code that would facilitate automatic assembly using a variety of values for those input parameters. This approach is similar to that used by Kansas State University in their program Stitch [16], except that it does not perform assembly refinement steps (see [15]), and it breaks each assembly into its component parts in order to reduce the computational resources required. We used our code to generate 405 unrefined *de novo* assemblies of our *Gossypium raimondii* dataset, each with a different combination of the input parameters shown in Table 1.

**Table 1:** Input parameter values

Parameter	Overlap significance threshold	False positive labels per 100 kbp	Proportion restriction sites unlabeled	Min. molecule length (kbp)	Min. labels per molecule
Values	1.11E-04	0.5	0.15	100	6
	1.11E-06	1.5	0.3	150	8
	1.11E-08	2.5	0.45	180	10
	1.11E-10				
	1.11E-12				

*Min.* is short for minimum

We assessed the quality of the assemblies based on their contiguity, their internal consistency, and their accuracy according to the reference genome. Assemblies were scored for total length, contig N50 length, and length of longest contig for contiguity. Internal consistency was divided into two metrics, the average number of overlapping molecules in which each label is observed, and the proportion of molecules not excluded from the assembly as singletons. Finally, we measured accuracy by comparing our assemblies to a highly contiguous reference genome sequence, using software provided by BNG. We report the weighted average confidence score, where confidence is the negative, 10-base logarithm of the *p*-value of an alignment.

## Results

### Data collection

We collected a total of 217.28 Gigabase-pairs (Gbp) of physical map data over nine, two-flow-cell runs of BNG’s Irys® machine. This is enough data for ~241× coverage of the similar to 900 Megabase-pair (Mbp) *G. raimondii* genome. Data statistics for individual flow cells are shown in Table 2. The weighted average across datasets for the molecule N50 length was 165.37 kbp. The expected label density using Nt.BspQ1 and Nt.BbvCl was 12.6 labels per 100 kbp. Our observed label density was consistently lower than the expected (max 11.3 labels per 100 kbp, weighted average 9.2).

**Table 2:** Map data collected

Date Run	Quantity (Mbp)	Molecule N50 (kbp)	Average labels per 100 kbp
28-May-14	5,861.00	218.6	7.2
04-Jun-14	15,723.90	154.5	8.2
05-Jun-14	32,131.70	150.4	8.6
05-Jun-14	18,135.40	143.9	9
22-Jul-14	7,122.50	188.7	6.1
23-Jul-14	9,651.20	175.8	9.3
24-Jul-14	2,833.90	165.8	9.1
24-Jul-14	5,492.80	198.6	10.2
25-Jul-14	15,037.10	189.7	6.1
28-Jul-14	6,246.70	189.7	6.6
29-Jul-14	4,848.80	155.4	10
30-Jul-14	9,029.30	163.8	10.1
31-Jul-14	15,970.40	168.3	10.1
05-Aug-14	12,213.10	171.2	10.3
06-Aug-14	15,718.60	169.8	10.2
07-Aug-14	7,312.50	161.5	10.6
07-Aug-14	1,176.00	155.5	11.3
07-Aug-14	17,104.90	160	10.9
07-Aug-14	15,670.10	150.6	11

### A tool for efficient trial and error

In order to generate a large number of assemblies in a reasonable amount of time, we developed the code OMWare, available at [17]. Our code generates a set of unrefined assemblies that results from a certain combination of input parameters. It automatically detects and runs only the minimum number of compatible precursor steps. Unrefined assembly with BNG’s software proceeds in four steps. First, input molecules are sorted. Second, they are split into files of approximately equal sizes, for computational efficiency. Third, each unique pair of molecules is aligned to produce an overlap score based on label pattern similarity. Finally, genomic regions are assembled using overlap scores in an overlap-layout-consensus graph. In order to produce 405 unrefined assemblies, OMWare performed only nine pairwise alignment steps, and a single split and sort step. The combinations of input parameters that affect certain steps, as well as the computational resource requirements of those steps, are shown in Table 3. The code also includes an interface to read and write data in the file formats used by BNG.

**Table 3:** Compute resources required for de novo assembly

Assembly step	Sort	Split	Pairwise alignment	Assembly	Total
Applicable parameters	Minimum length^a^, minimum labels^a^	Minimum length^a^, minimum labels^a^	Minimum length^a^, minimum labels^a^, significance threshold^a^, false positive, false negative	Minimum length, minimum sites, significance threshold, false positive, false negative	Minimum length, minimum sites, significance threshold, false positive, false negative
Steps run	1	1	9	405	-
Parallel jobs per step	1	3	1,250	1	-
Minutes elapsed	1	6	3,442,500	105,614	3,548,121
Predicted^b^ minutes	405	2,733	154,912,500	105,614	155,021,252
Megabytes RAM used	1	5,667	176,321,250	1,130,838,484	1,307,165,402
Predicted^b^ megabytes	405	2,295,135	7,934,456,000	1,130,838,484	9,067,590,024
Megabytes disk space used	580	580	4,640,000	51,874	4,693,034
Predicted^b^ megabytes	2,900	2,900	208,800,000	51,874	209,089,674

^a^Input parameter applies only as an output filter; it does not affect the algorithms internal workings. A step run with lenient parameters can serve as input for a more stringent downstream step, which will filter its input

^b^Estimation of resources required if all 405 Sort, Split, and Pairwise alignment steps were run

### Assembly quality

Contiguity and internal consistency varied widely between assemblies, and were predominantly controlled by two input parameters, minimum molecule length and significance threshold. The maximum total length of any assembly was about 1.1 Gbp, which is larger than the expected genome size of about 900 Mbp. The shortest assembly covered only 78 Mbp. Contig N50 lengths ranged from 252 to 431 kbp, and the maximum length of any single contig was 2.24 Mbp. In every assembly, a large proportion of input molecules, from 0.90 to 0.993, were excluded as singletons. Across parameter combinations, the average number of molecules in which each label was observed was between five and nine.

**Fig. 1:**
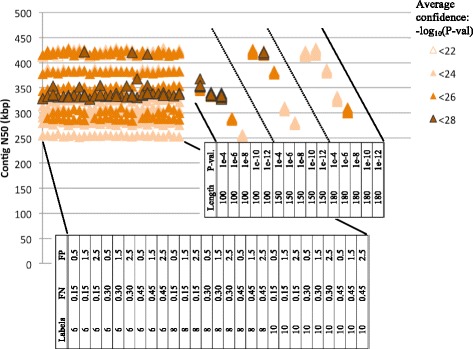
Assembly accuracy and contig N50 lengths are affected by different input parameters. Contig N50 lengths are relatively stable to permutations of false positive label rates (FP, per 100 kbp), false negative label rates (FN), and minimum labels per molecule (Labels) (*left*). When the same assemblies are grouped by minimum molecule length (Lengths, in kbp) and significance threshold (*P*-val.) (*right*), more substantial changes in response to these input parameters are observed. Some inaccurate assemblies have high N50 lengths. The converse is also true

**Fig. 2:**
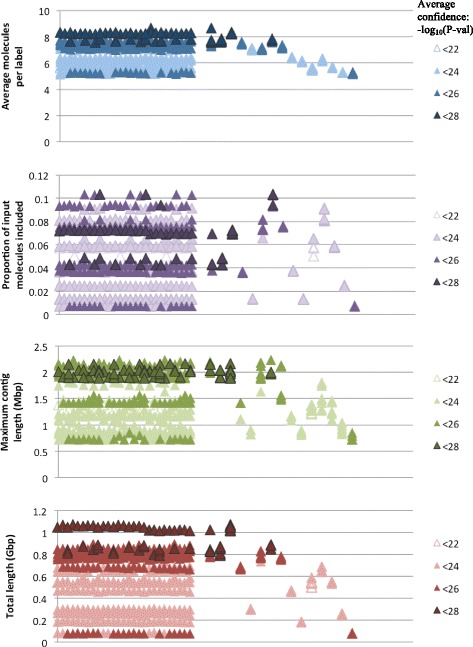
Several metrics of assembly contiguity and internal consistency fail to predict assembly accuracy

The accuracy of assembled contigs also varied, and appeared to correspond very little with measures of contiguity or internal consistency. The lowest average confidence score of any assembly was 21.4 (*p*-value ≈ 3.9e-22), and the highest was 27.8 (*p*-value ≈ 1.5e-28). There were no outliers in confidence. The confidence scores are more responsive to changes in false positive and false negative label rates than metrics of contiguity appear to be. The distribution of both contiguity and confidence scores as they are affected by various input parameters can be seen in Figs. 1 and 2.

## Discussion and conclusions

A major limitation of this study was data quality. In the data, we observed label densities that were consistently lower than expected. It may be worth noting that BNG recommends keeping false positive and false negative label rates below 1.5 erroneous labels per 100 kbp and 0.15 of restriction sites unlabeled. Even with very conservative false positive label rate estimates, our data has a false negative label rate above this recommended threshold. This may be due to the inherent difficulty of extracting contaminant free HMW DNA from plants [18]. Additionally, it is reasonable to suspect that by using two nicking enzymes simultaneously in a buffer that was optimized for only one of the two, we inadvertently reduced enzyme efficiency. These factors may help explain the low internal consistency observed in our assemblies.

It is clear that OMWare is far more efficient than the BNG software at generating a large number of unrefined assemblies. Trial and error showed promise for use in scenarios when no reference genome is available. However, without using a reference genome, we were unable to detect reliable, genome-independent predictors of assembly quality in the five metrics of contiguity and internal consistency that we examined.

In scenarios where a reference genome is available, alternative software for empirical estimation of input parameters exists and requires fewer computational resources than OMWare. In the case of BNG’s software, there are some input parameters that OMWare assays that their software does not, such as minimum sites per molecule, minimum molecule length, and significance threshold. There are also input parameters that OMWare does not test. Molecule stretch or stain inconsistencies manifest as variable distances observed between labels. We did not incorporate this into OMWare because BNG’s internal assembly algorithm uses three separate parameters to compensate for variable distances, permutations on which would have substantially increased the necessary compute resources.

This analysis does yield some interesting insight into the behavior of the *de novo* assembly algorithm. For example, contiguity and internal consistency change far more in response to significance thresholds and minimum molecule lengths, and by extension, coverage, than they do to false positive and false negative label rates. However, assembly accuracy, as measured by significance of alignments to the reference genome, does respond to these assumptions about enzyme efficiency.

## Abbreviations

BNG, BioNano Genomics; Gbp, gigabase-pairs; HMW, high molecular weight; kbp, kilobase-pairs; Mbp, megabase-pairs; MPS, massively parallel sequencing.
